# The Long Noncoding RNA, LOC645166, in T Cells of Ankylosing Spondylitis (AS) Patients Regulates the FOXP3 Expression via the Axis of LOC645166/miR-188-5p/NFKBID

**DOI:** 10.1155/mi/8574340

**Published:** 2025-09-15

**Authors:** Hui-Chun Yu, Kuang-Yung Huang, Ming-Chi Lu, Hsien-Yu Huang Tseng, Ning-Sheng Lai, Hsien-Bin Huang

**Affiliations:** ^1^Department of Medical Research, Dalin Tzu Chi Hospital, Buddhist Tzu Chi Medical Foundation, Chiayi 62247, Taiwan; ^2^Division of Allergy, Immunology and Rheumatology, Department of Medicine, Dalin Tzu Chi Hospital, Buddhist Tzu Chi Medical Foundation, Chiayi 62247, Taiwan; ^3^School of Medicine, Tzu Chi University, Hualien 970, Taiwan; ^4^Department of Biomedical Sciences, National Chung Cheng University, Chiayi 621, Taiwan

## Abstract

The expression of long noncoding RNA (LncRNA), LOC645166, is downregulated in T cells of ankylosing spondylitis (AS) patients. The role of LOC645166 in contribution to AS pathogenesis was investigated. Here, we have identified that an interacting network of LOC645166/miR-188-5p/NF-κB inhibitor-D (NFKBID) occurs in T cells of AS patients and regulates the expression of forkhead box P3 (FOXP3). Downregulation of LOC645166 augments the levels of miR-188-5p that binds to the 3′-UTR of NFKB1D mRNA and blocks the NFKB1D expression. NFKB1D, also called IκB_NS_, can trigger regulatory T (Treg) cell development through induction of FOXP3 expression. Downregulation of NFKB1D expression leads to suppression of the FOXP3 induction, in turn affecting Treg cell development. Promotion of the autoimmune response induced by suppression of FOXP3 expression contributes to the pathogenesis of AS.

## 1. Introduction

Ankylosing spondylitis (AS) is one type of arthritic diseases that is characterized by a chronic inflammation in joints of spine. The bones in the spine of some AS patients will develop to fusion, resulting in ankylosis and syndesmophytes with time [1–4]. People that carry the HLA-B27 gene have the higher risk to develop AS [5, 6]. Indeed, more than 95% of AS patients carry the HLA-B27 gene. However, only 1%–2% of the HLA-B27 positive population will develop AS, suggesting that other genetic or environmental factors may also contribute to the development of AS [1–4].

Long noncoding RNAs (LncRNAs) are transcripts with more than 200 bases in length, but they cannot be translated into proteins. LncRNAs can modulate gene expression via different programs, such as alteration of epigenetic, transcriptional, or post-transcriptional regulation [7]. A growing body of evidence has revealed that LncRNAs make the contributions to the development of AS [8–10]. Dysregulation of LncRNAs has been observed in AS, resulting in upregulating the signaling of proinflammatory cytokines and promoting the activity of IL-23/IL-17 axis. These augmentations enhance the inflammation and aberrant bone formation [10]. Recently, we have demonstrated that the levels of LOC645166 in T cells of AS patients were downregulated [11]. Overexpression of LOC645166 in Jurkat cells or T cells isolated from AS patients reduces the STAT3 activation upon stimulation with anti-CD3/CD28 antibodies. LOC645166 prefers binding to the K63-linked polyubiquitin chains and blocks the recruitment of IKK complex onto these chains, in turn suppressing the IKK2 activation by transautophosphorylation or phosphorylation by TAK1 of TAK1/TAB2/3 complex. Activation of IKK2 is essential for NF-κB activation [12–17]. NF-κB is associated with IκBs to form an inactive complex that is retained in the cytoplasm. IκBs is phosphorylated by the activated IKK2. After phosphorylation, IκBs is targeted for modification by the K48-linked polyubiquitin reaction and then is degraded by proteasome. Without inhibition by IκBs, NF-κB will be translocated into the nucleus and regulate the expression of targeted genes. Thus, downregulation of LOC645166 in T cells of AS patients enhances the sensitivity of NF-κB signaling stimulated by the proinflammatory cytokines or by TLR ligands [11].

Regulatory T (Treg) cells are important mediators of immune system, regulating maintenance of self-antigen tolerance and immune homeostasis [18, 19]. Treg cells suppress activation and proliferation of effector T cells and prevent the autoimmunity disease. Treg cells are classified as the induced Treg (iTreg) cells and natural Treg (nTreg) cells, both of which are derived from development in different lymphoid tissues. iTreg cells differentiate from naïve T cell precursors in peripheral lymphoid tissues. nTreg cells develop in the thymus [20–24]. Treg cells are characterized by expression of CD4, CD25, and forkhead box P3 (FOXP3) [23–25]. FOXP3, a forkhead transcription factor, is a critical regulator that dominates the development and phenotypic maintenance of Treg cells. Several lines of evidence have revealed that nTreg cell and iTreg cell development are driven by NF-κB signaling [26–28]. Indeed, NF-κB directly binds to the promoter, CNS2, and CNS3 within the *FOXP3* locus and drives FOXP3 expression [23, 26–31]. It has been known that NF-κB inhibitor-D (NFKBID) also plays a critical role in regulation of FOXP3 expression under activation of NF-κB signaling [32]. NFKBID (also called IκB_NS_) belonging to a member of BCL-3 subfamily is one of the atypical IκB proteins [33] and serves as a transcriptional enhancer or repressor via association with NF-κB transcription factors. It binds to the promoter and CNS3 with NF-κB at the *FOXP3* locus and promotes expression of FOXP3 [32]. NFKBID is stable, highly induced, and not degraded by proteasome after NF-κB signaling [34, 35].

In the current study, we explored the biological roles of LOC645166 in regulation of AS pathogenesis. We identified an interaction network of LOC645166/miR-188-5p/NFKBID present in T cells of AS patients. This interaction axis regulates the expression of FOXP3, affecting the development of Treg cells in T cells of AS patients.

## 2. Materials and Methods

### 2.1. Materials

Acrylamide, N,N′-methylenebisacrylamide, ammonium persulfate, TEMED, Tris base, 2-mercaptoethanol, dithiothreitol, sodium dodecyl sulfate (SDS), glycine, and NP-40 were purchased from Sigma-Aldrich (St. Louis, MO, USA). Anti-CD3, anti-CD28, anti-actin, anti-NFKBID, and anti-FOXP3 were ordered from Cell Signaling Technology (Danvers, MA, USA). miR-188-5p, miRNA mimic negative control, miR-188-5p inhibitor, and miRNA inhibitor negative control were purchased from Thermo Fisher Scientific (Waltham, MA, USA).

### 2.2. Quantitation of miR-188-5p and NFKBID mRNA in T Cells by qRT-PCR

Human PBMCs, T cells, or total RNAs of T cells isolated from the AS patients or healthy controls followed the methods as described [11]. Quantitation of miR-188-5p and NFKBID mRNA followed the method as described by Yang et al. [36] and Hirotani et al. [37], respectively. U6 was used as the internal control in the detection of miR188-5p. The following primers were used: miR-188-5p forward, 5′- CCC TCT CTC ACA TCC CTT GCA T -3′ and reverse, 5′- ATC CTG CAA ACC CTG CAT GTG -3′; U6 forward, 5′-CTC GCT TCG GCA GCA CAT ATA CT-3′ and reverse, 5′-ACG CTT CAC GAA TTT GCG TGTC-3′; NFKBID forward, 5′-GCT GTA TCC TGA GCC TTC CCT GTC-3′ and reverse, 5′-GCT CAG CAG GTC TTC CAC AAT CAG-3′; β-actin forward, 5′-CTA TGT GGG TGA CGA GGC CCA GAG-3′ and reverse 5′-GGG TAC ATG GTG GTA CCA CCA GAC-3′. The relative expression ratio of miR-188-5p and NFKBID mRNA was normalized to U6 and to β-actin, respectively, and was quantified using the 2^−ΔΔCq^ method.

### 2.3. The Effects of LOC645166 Overexpressed in Jurkat Cells on the Expression Levels of miR-188-5p or LOC645166

Construction of pcDNA3.1-LOC645166 vector, transfection of Jurkat cells (1.5 × 10^6^ cells) with pcDNA3.1-LOC645166 (1 μg) or with pcDNA3.1 (1 μg) by using electroporation and maintenance of the transfected cells followed the method described by Yu et al. [11]. After 24 h, the transfected cells were harvested by centrifugation. Quantitation of miR-188-5p and LOC645166 followed the abovementioned method.

### 2.4. miRNA Coimmunoprecipitation

miRNA IP was carried out by miRNA Target IP kit (Catalog Number 25500, Active Motif, Carlsbad, California, USA). Jurkat cells (3 × 10^6^ cells) were seeded in petri dish (10 cm) and cultured in RPMI-1640 medium with 10% fetal calf serum (FCS) and 5% CO_2_ at 37°C overnight, followed by transfection with miR-188-5p (5 μM) using electroporation. After incubation at 37°C for 24 h, the RISC complex was immunoprecipitated by using the Ago1/2/3 antibodies, following the methods as described by the manufacturer. The precipitated products, LOC645166, and NFKBID mRNA, were analyzed by qRT-PCR.

### 2.5. Western Blotting

Jurkat cells (1.5 × 10^6^ cells) were transfected with pc-DNA3.1-LOC645166 (1 μg), miR-188-5p (5 μM) or miR-188-5p inhibitor (5 μM) by electroporation. The transfected cells were maintained in RPMI-1640 medium with 10% FCS at 37°C with 5% CO_2_ plus antibiotics (100 IU/mL of penicillin and 100 μg/mL of streptomycin). After 48 h, cells were harvested by centrifugation. The pelleted cells were resuspended in 100 μL of 1% SDS and ruptured by ultrasonication. An aliquot of lysate (50 μg) was analyzed by western blotting, probed for actin or NFKBID. To detect the expression levels of FOXP3, the transfected Jurkat cells were activated by PMA (20 ng/mL) and ionomycin (500 ng/mL) for 48 h. The expressed FOXP3 was detected by western blotting. To analyze the protein levels of NFKBID in T cells or FOXP3 in CD4^+^ T cells of AS patients or healthy controls, the isolated cells from both groups were resuspended in 0.1% SDS (100 μL) and ruptured by ultrasonication. An aliquot of extracted proteins (50 μg) was analyzed by western blotting, probed for actin, NFKBID, or FOXP3. To examine the effect of NFKBID on the expression of FOXP3, the NFKBID mRNA of Jurkat cells was knocked down by using siRNA (sc-97928, Santa Cruz Biotechnology). Jurkat cells (1.5 × 10^6^ cells) were transfected with siRNA (1 μg) by electroporation. The transfected cells were treated with PMA (20 ng/mL) and ionomycin (500 ng/mL) and maintained in RPMI-1640 medium as the abovementioned method. After 48 h, cells were harvested by centrifugation. The pelleted cells were resuspended in 100 μL of 0.1% SDS and ruptured by ultrasonication. An aliquot of extracted proteins (50 μg) was analyzed by western blotting, probed for actin, NFKBID, or FOXP3.

### 2.6. Flow Cytometry

CD4^+^ T cells isolated from PBMCs of AS patients or healthy controls were washed with PBS three times and stained with BB515-conjugated anti-human CD25 antibody (BD Life Sciences, San Jose, CA, USA.Cat. #565096; 1:500 dilution) in the dark for 30 min. The stained cells were washed with PBS three times, fixed, and permeabilized by Human FOXP3 Buffer Set (BD Life Sciences, San Jose, CA, USA, Cat. #560098). Then, the treated cells were incubated with PE-conjugated anti-human FOXP3 mAb (BD Life Sciences, Cat. #560046; 1:500 dilution) in the dark for 30 min, washed with PBS three times, and analyzed by flow cytometry.

### 2.7. Ethics Statement

AS patients were defined according to the modified New York criteria [38]. All involved AS patients and healthy controls enrolled from August 2021 to July 2023 in Buddhist Dalin Tzu Chi General Hospital, Chiayi, Taiwan, and signed an informed consent form approved by the Institutional Review Board and Ethics Committee (IRBEC). The protocol for isolation of human T cells or CD4^+^ T cells from the participant's PBMCs has been reviewed and approved by IRBEC (B11003004). Basic characteristics of the AS patients are shown in Supporting Information Table SI.

### 2.8. Statistical Analysis

The mean ± standard deviation (SD) represents the data obtained from at least three independent experiments. The Mann–Whitney *U* test was used to analyze statistical significance which was set at *p*  < 0.05.

## 3. Results

### 3.1. Identification of a LOC645166/mR-188-5p/NFKBID Interacting Network

**Figure 1:**
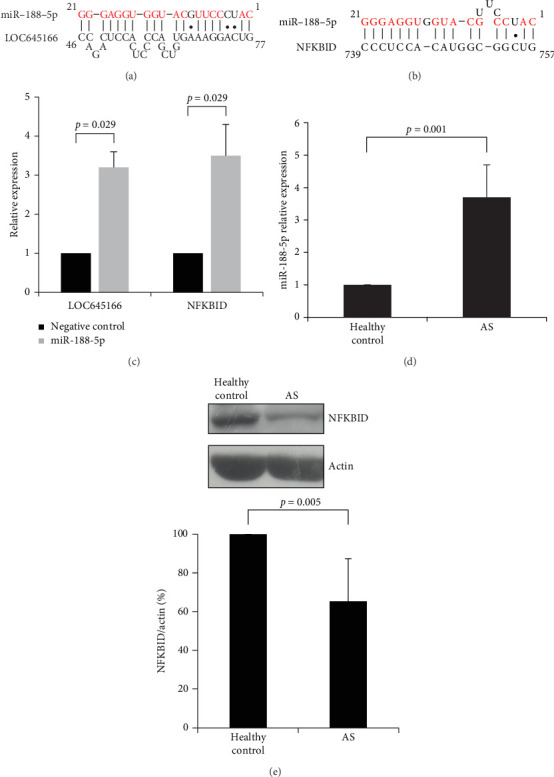
The LOC645166/miR-188-5p/NFKBID interaction network is present in T cells of AS patients. (A) LOC645166 hybridizes with miR-188-5p. (B) miR-188-5p hybridizes with the 3′-UTR of NFKBID mRNA. (C) The effect of miR-188-5p overexpressed in Jurkat cells on LOC645166 and NFKBID mRNA coimmunoprecipitated by anti-Ago1/2/3 antibodies (*N* = 4). (D) The expression levels of miR-188-5p in T cells of AS patients and healthy controls (*N* = 13). (E) The analysis of NFKBID protein in T cells of AS patients and healthy controls by the western blotting (*N* = 5).

LncRNAs can serve as a sponge to affinity with miRNAs and block the lncRNA-targeted miRNA to associate with its targeted mRNA, leading to altering the expression of miRNA-targeted mRNAs and in turn regulating the cell functions. In this study, we focused on the LOC645166-associated miRNAs that can regulate the inflammation and immune response. We have searched the LOC645166-associated miRNAs by using the website of lncRABKB (https://ngdc.cncb.ac.cn/lncbook/). Many LOC645166-associated miRNAs, including miR-188-5p, hsa-miR-924, hsa-miR-891b, hsa-miR-770-5p, hsa-miR-767-3p, hsa-miR-766-3p, hsa-miR-661, and hsa-miR-645, were identified. One miRNA (miR-188-5p) that may regulate the immune response and inflammation attracted our attention. The site for miR-188-5p to associate with LOC645166 is shown in Figure 1A. We searched the possible mRNAs targeted by miR-188-5p and identified that the 3′-UTR of NFKBID mRNA is targeted by miR-188-5p (Figure 1B). We verified whether the LOC645166 and NFKBID mRNA were targeted by miR-188-5p. Jurkat cells were transfected with a miR-188-5p or a nontargeting miRNA control for 24 h. Immunoprecipitation was carried out by using anti-Ago1/2/3 antibodies and the precipitated products were analyzed by quantitative RT-PCR to verify whether the levels of LOC645166 or NFKBID mRNA were increased in the RISC complex after miR-188-5p was transfected into Jurkat cells. The result shown in Figure 1C reveals that the levels of LOC645166 and NFKBID mRNA were increased in the RISC complex, indicating both of which were targeted by miR-188-5p. Compared with the healthy controls, the levels of LOC645166 were downregulated in T cells of AS patients [11]. Reduction of association with LOC645166 suggested that the levels of miR-188-5p and the expression of NFKBID mRNA targeted by miR-188-5p will be increased and decreased in T cells of AS patients, respectively. Indeed, compared with the healthy controls, the levels of miR-188-5p (Figure 1D) and NFKBID protein (Figure 1E) in T cells of AS patients are augmented and declined, respectively.

### 3.2. Overexpression of LOC645166 in Jurkat Cells Suppresses the Expression Levels of miR-188-5p and Promotes the NFKBID Production

**Figure 2:**
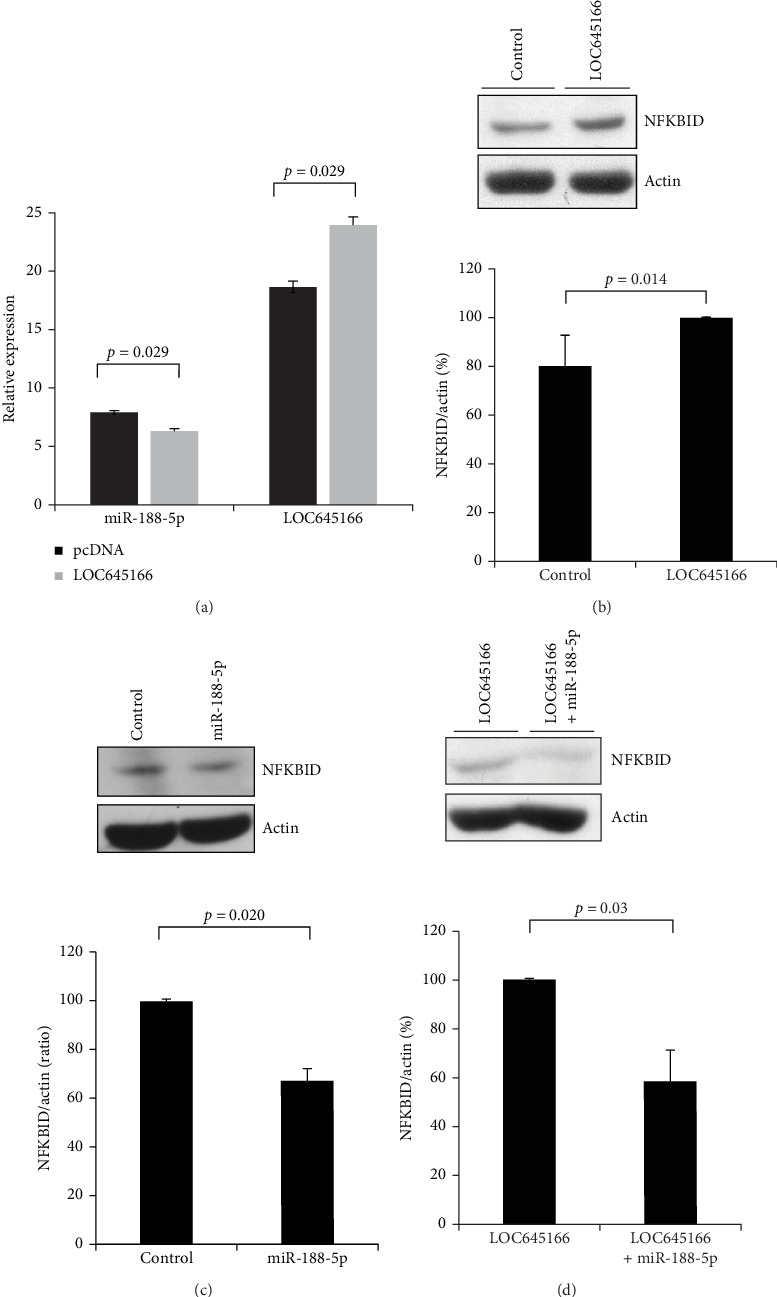
The effect of LOC645166 overexpressed in Jurkat cells on the levels of miR-188-5p and NFKBID protein. (A) The effect of LOC645166 overexpressed in Jurkat cells on the levels of miR-188-5p (*N* = 4). (B) The effect of LOC645166 overexpressed in Jurkat cells on the protein levels of NFKBID (*N* = 4). (C) The effect of miR-188-5p overexpressed in Jurkat cells on the protein levels of NFKBID. (D) The effect of LOC645166 co-overexpressed with miR-188-5p in Jurkat cells on the protein levels of NFKBID (*N* = 4).

To verify the interacting network of LOC645166/miR-188-5p/NFKBID, LOC645166 was overexpressed in Jurkat cells and the expressed levels of miR-188-5p and NFKBID protein were examined. The results show that overexpression of LOC645166 significantly reduces the expression of miR-188-5p (Figure 2A) but upregulates the production of NFKBID protein (Figure 2B). Overexpression of miR-188-5p in Jurkat cells downregulates the production of NFKBID (Figure 2C). If the expression vectors, LOC645166 and miR-188-5p, were codelivered into Jurkat cells by electroporation, the expression of NFKBID proteins was suppressed (Figure 2D), indicating that the NFKBID mRNA was targeted by miR-188-5p.

### 3.3. Treatment With miR-188-5p Inhibitor Enhances the Expression of NFKBID Protein

**Figure 3:**
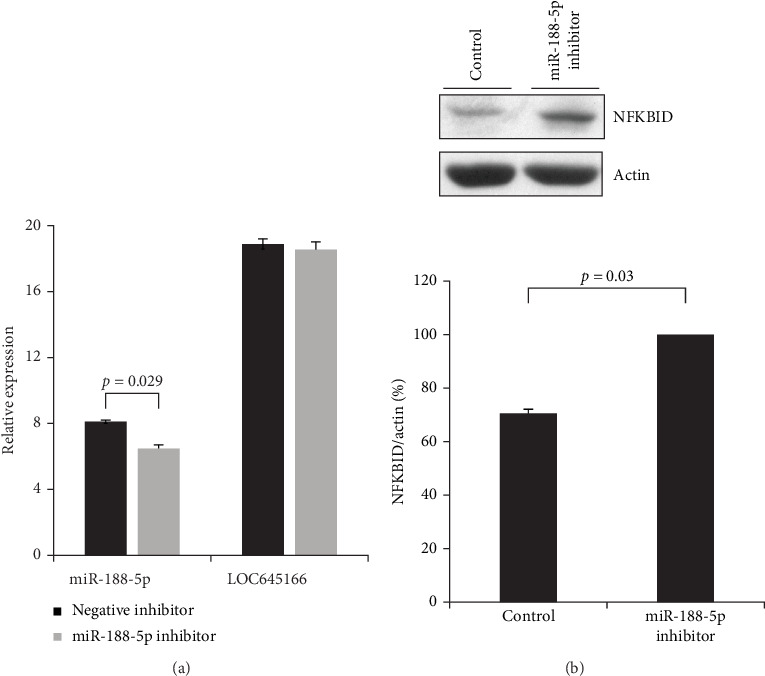
The effect of miR-188-5p inhibitor electro-transferred in Jurkat cells on the levels of miR-188-5p and of NFKBID protein. (A) The effect of miR-188-5p inhibitor electro-transferred in Jurkat cells on the levels of miR-188-5p (*N* = 4). (B) The effect of miR-188-5p inhibitor electro-transferred in Jurkat cells on the protein levels of NFKBID (*N* = 4).

The sequence of miR-188-5p inhibitor can anneal with miR-188-5p by antiparallel pairing and enhance the degradation of miR-188-5p after pairing with its inhibitor in cells. We delivered the miR-188-5p inhibitor into Jurkat cells by electroporation. The levels of miR-188-5p were analyzed by quantitative RT-PCR. Apparently, the expression levels of miR-188-5p were reduced after transfection with its inhibitor, but this treatment cannot affect the levels of LOC645166 (Figure 3A). In addition, a decrease in levels of miR-188-5p by treatment with miR-188-5p inhibitor increases the protein expression of NFKBID (Figure 3B).

### 3.4. The Expression of FOXP3 is Regulated by the Axis of LOC645166/miR-188-5p/NFKBID

**Figure 4:**
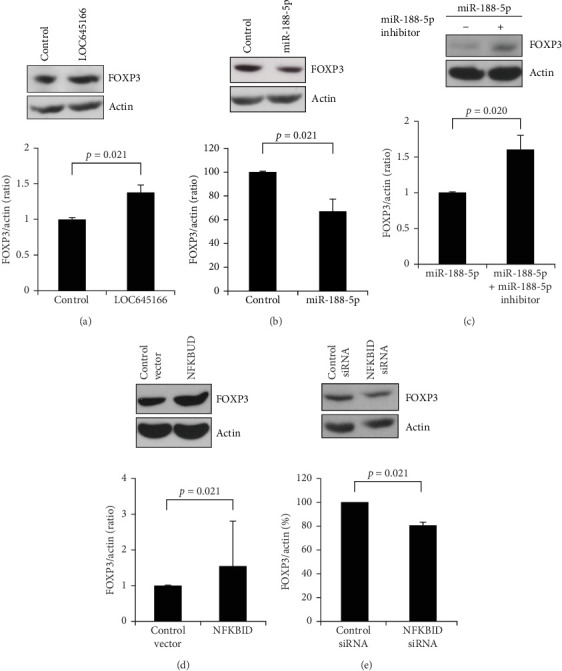
The effect of LOC645166/miR-188-5p/NFKBID axis on the protein levels of FOXP3 in Jurkat cells. (A) The effect of LOC645166 overexpressed in Jurkat cells on the protein levels FOXP3 (*N* = 4). (B) The effect of miR-188-5p electro-transferred in Jurkat cells on the FOXP3 expression (*N* = 4). (C) The effect of miR-188-5p co-transfected with its inhibitor in Jurkat cells on the protein levels of FOXP3 (*N* = 4). (D) The effect of NFKBID overexpressed in Jurkat cells on protein levels of FOXP3 (*N* = 4). (E) The effect of NFKBID knocked down by siRNA on the protein levels of FOXP3 (*N* = 4).

NFKBID belonging to a member of BCL-3 subfamily is one of the atypical IκB proteins [33]. The recent study has revealed that NFKBID binds to the promoter and CNS 3 of the *FOXP3* locus and drives the expression of FOXP3 under NF-κB signaling, in turn mediating Treg cell development [32]. Thus, we verified whether the axis of LOC645166/miR-188-5p/NFKBID regulates FOXP3 expression. Overexpression of LOC645166 in Jurkat cells promoted the expression of FOXP3 (Figure 4A). Overexpression of miR-188-5p in Jurkat cells suppressed the expression of FOXP3 (Figure 4B), while coexpression of miR-188-5p with its inhibitor attenuated the suppression and increased the FOXP3 expression (Figure 4C). In addition, overexpression and knockdown of NFKBID in Jurkat cells upregulated and restrained the expression of FOXP3, respectively (Figure 4D,E), suggesting that the expression of FOXP3 was modulated by the axis of LOC645166/miR-188-5p/NFKBID.

### 3.5. Downregulation of FOXP3 Expression in T Cells and Low Levels of Treg Cells in PBMCs of AS Patients

**Figure 5:**
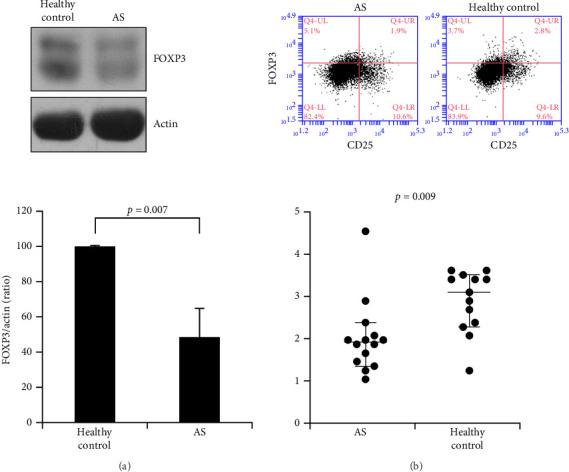
The expression levels of FOXP3 and Treg cell frequencies in CD4^+^ T cells of AS patients and healthy control. (A) The levels of FOXP3 in CD4^+^ T cells of AS patients and healthy controls (*N* = 5). (B) Flow cytometry analysis of the Treg cell frequencies in CD4^+^ T cells of AS patients (*N* = 14) and healthy controls (*N* = 13).

Downregulation of LOC645166 expression results in upregulation of miR-188-5p expression and reduction of NFKBID protein in T cells of AS patients (Figure 1D–F). Thus, we would like to examine whether the expression levels of FOXP3 protein are downregulated due to downregulation of NFKBID protein in T cells of AS patients. Indeed, compared with the healthy control group, the levels of FOXP3 in T cells of AS patients were significantly reduced (Figure 5A). FOXP3 is a key regulator in development of Treg cells [22–25]. Thus, we analyzed the levels of Treg cells (CD25^+^/FOXP3^+^) in T cells (CD4^+^) isolated from AS patients and healthy controls by flow cytometry. Compared with the healthy controls, the levels of Treg cells of AS patients were dramatically reduced (Figure 5B).

## 4. Discussion

The NF-κB signaling is a key positive regulator of Treg cell development via upregulation of FOXP3 induction [23, 26–31]. However, AS characterized as a chronic inflammatory disease exhibits a high activity of NF-κB signaling, but the FOXP3 induction and Treg cell development are suppressed in AS [39–41]. Compared with the healthy controls, AS patients display the low levels of CD4^+^CD25^+^FOXP3^+^ Treg cells (Figure 5B). In this study, we found the molecular mechanism for suppression of Treg cell development and the FOXP3 induction in AS patients. Downregulation of LOC645166 in T cells of AS patients plays a critical role in upregulation of NF-κB signaling and simultaneous downregulation of FOXP3 induction, leading to suppression of Treg cell development. LOC645166 that regulates the NF-κB signaling pathway arises from acting as a protein sponge to preferably associate with K63-linked polyubiquitin chains and impedes the recruitment of IKK complex onto these chains, in turn blocking the IKK2 activation and suppressing the NF-κB activation [11–17]. The low levels of LOC645166 in T cells of AS patients will enhance the NF-κB signaling after stimulation by proinflammatory cytokines or ligands of toll-like receptor.

To mediate Treg cell development, LOC645166 serves as a sponge to anneal with miR-188-5p, rendering the LOC645166/miR-188-5p complex to be degraded and downregulating the levels of miR-188-5p. The 3′-UTR of NFKBID mRNA is the target site by miR-188-5p. NFKBID that has been known to play an important role in regulation of FOXP3 induction is an atypical IκB protein and is temporarily expressed during nTreg cell development [32]. NFKBID binds to the promoter and CNS3 of *FOXP3* locus via association with NF-κB (p50 or c-Rel) and induces the expression of FOXP3, in turn mediating Treg cell development. NFKBID-deficient mice display an augmentation of GITR^+^CD25^+^FOXP3^−^ Treg precursor cells and a reduction of around 50% mature Treg cells attributable to malfunctional FOXP3 induction. NFKBID does not alter the phenotypic function of the mature Treg cells [32]. Here, we found that the FOXP3 protein expression was reduced and augmented when Jurkat cells were overexpressed with miR-188-5p and NFKBID, respectively (Figure 4C,D). Thus, downregulation of LOC645166 of T cells in AS patients results in an increased level in miR-188-5p and a decreased level in NFKBID and FOXP3 induction, leading to suppressing Treg cell development. The network interaction induced by downregulation of LOC645166 can account for the situation where the levels of CD4^**+**^CD25^**+**^FOXP3^**+**^ Treg cells of AS patients are lower than those of healthy controls even though the NF-κB activity is highly promoted in AS patients.

Treg cells restrain immune responses via adjusting the activity of effector immune cells and maintain immunological self-tolerance to avoid autoimmune diseases [19, 20]. The characteristic of Treg cells expresses CD4, CD25, and FOXP3. FOXP3 is a critical regulator that controls maintenance and development of Treg cells [23–25]. Mutations in *FOXP3* can cause a functional loss of Treg cells, leading to a severe autoimmune disease in human and mouse [42, 43]. The NF-κB signaling is induced by TCR engagement during nTreg cell development [44, 45]. The signaling pathway of TCR engagement to activate NF-κB activity is sophisticated [46, 47]. TCR-mediated signals and CD28 costimulation trigger recruitment of PKCθ on the membrane to phosphorylate the adaptor protein, CARMA1. Phosphorylation of CARMA1 induces its conformational change and initiates the assembly of a filamentous CARMA1–BCL10–MALT1 (CBM) complex. MALT1 in this signaling hub can enroll the E3 ligase, TRAF6, which then catalyzes the K63-linked polyubiquitin synthesis on itself, BCL10, and MALT1. These K63-linked polyubiquitin chains function as docking sites for TAB2/3 of TAK1-TAB2/3 complex or NEMO of the IKK complex, in turn initiating the IKK2 activation and then triggering the activation of NF-κB signaling. The active NF-κB is translocated into nucleus, binds to the promotor, CNS2 and CNS3 of *FOXP3* locus and promotes the expression of FOXP3 [23, 26–31]. Although downregulation of LOC645166 in T cells promotes the NF-κB signaling during TCR engagement, the FOXP3 induction is restrained via suppression of NFKBID by the increased levels of miR-188-5p. Taken together, LOC645166 carries out the different routes to simultaneously regulate the proinflammatory reaction and immune response. Downregulation of LOC645166 in T cells of AS patient promotes the NF-κB signaling but suppresses the FOXP3 induction and Treg cell development via regulation of LOC645166/miR-188-5p/NFKBID axis.

A reduction in the protein level of FOXP3 in Treg cells isolated from PBMCs of active AS patients has also been found by Guo et al. [48]. This reduction of FOXP3 protein is linked with active AS and affects the functions of Treg cells, including being incapable of completely repressing naïve T cell proliferation, displaying abnormal IL-2 signaling, and CNS2 hypermethylation in the *FOXP3* gene locus, unable to effectually carry out IL-2 signaling and exhibiting relatively lower STAT5 phosphorylation in response to IL-2 stimulation. The defective functions of Treg cells make the contribution to AS pathogenesis and provide a potential therapeutic target for AS treatment.

## 5. Conclusion

The interaction axis of LOC645166/miR-188-5p/NFKBID occurs in T cells of AS patients. Thus, downregulation of LOC645166 expression in T cells of AS patients promotes the expression of miR-188-5p and in turn suppresses the expression of NFKBID to impede the expression of FOXP3, accounting for reduction of Treg cell development in AS patients.

## Data Availability

The data used in the current study are available upon request.

## Supporting Information

Supporting Information Table SI. Basic characteristics of the AS patients.
