# Supplementary material for: The Long Noncoding RNA, LOC645166, in T Cells of Ankylosing Spondylitis (AS) Patients Regulates the FOXP3 Expression via the Axis of LOC645166/miR-188-5p/NFKBID
Source: Mediators Inflamm. 2025 Sep 15;2025:8574340. doi: 10.1155/mi/8574340 (PMC12453897; doi:10.1155/mi/8574340)

**Supplementary Table I for:**

**The long noncoding RNA, LOC645166, in T cells of ankylosing spondylitis (AS) patients regulates the FOXP3 expression via the axis of LOC645166/miR-188-5p/NFKBID**

Hui-Chun Yu^1,^*, Kuang-Yung Huang^2,3^, Ming-Chi Lu^1,2,3^, Hsien-Yu Huang Tseng^1^, Ning-Sheng Lai^2,3^*, and Hsien-Bin Huang^4,^*

^1^Department of Medical Research, Dalin Tzu Chi Hospital, Buddhist Tzu Chi Medical Foundation, Chiayi, 62247, Taiwan

^2^Division of Allergy, Immunology and Rheumatology, Department of Medicine, Dalin Tzu Chi Hospital, Buddhist Tzu Chi Medical Foundation, Chiayi, 62247, Taiwan

^3^School of Medicine, Tzu Chi University, Hualien 970, Taiwan

^4^Department of Biomedical Sciences, National Chung Cheng University, Chiayi 621, Taiwan


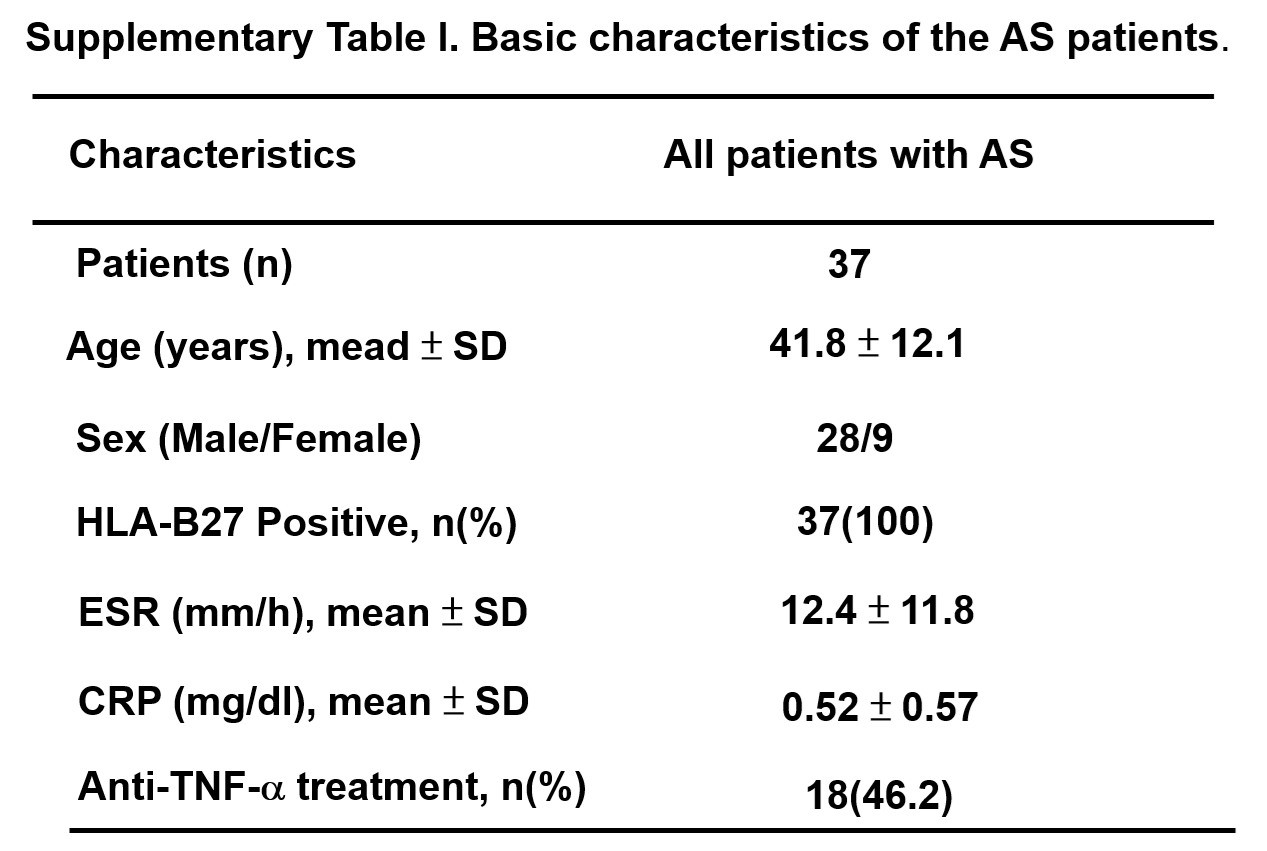

Supplement: Supporting Information — Table SI. Basic characteristics of the AS patients. [file 8574340.f1.docx]
